# Long terms trends in CD4^+^ cell counts, CD8^+^ cell counts, and the CD4^+^ : CD8^+^ ratio

**DOI:** 10.1097/QAD.0000000000001848

**Published:** 2018-06-01

**Authors:** Rachael A. Hughes, Margaret T. May, Kate Tilling, Ninon Taylor, Linda Wittkop, Peter Reiss, John Gill, Philipp Schommers, Dominique Costagliola, Jodie L. Guest, Viviane D. Lima, Antonella d’Arminio Monforte, Colette Smith, Matthias Cavassini, Michael Saag, Jessica L. Castilho, Jonathan A.C. Sterne

**Affiliations:** a Population Health Sciences, Bristol Medical School; b MRC Integrative Epidemiology Unit, University of Bristol, Bristol, UK; c Department of Internal Medicine III, Paracelsus Medical University, Salzburg, Austria; d Team MORPH3EUS, Bordeaux Population Health Research Center, University Bordeaux, ISPED, INSERM, UMR 1219, CIC-EC 1401; e Service d’Information Medicale, Pole de Sante Publique, CHU de Bordeaux, Bordeaux, France; f Stichting HIV Monitoring; g Division of Infectious Diseases, Academic Medical Center, University of Amsterdam; h Department of Global Health, Academic Medical Center, University of Amsterdam; i Amsterdam Institute for Global Health and Development, Amsterdam, The Netherlands; j Division of Infectious Diseases, University of Calgary, Calgary, Alberta, Canada; k Department I of Internal Medicine, University Hospital of Cologne, Cologne, Germany; l Institut Pierre Louis d’Épidémiologie et de Santé Publique (IPLESP), INSERM, Sorbonne Université, Paris, France; m HIV Atlanta Veterans Affairs Cohort Study, Atlanta Veterans Affairs Medical Center, Decatur, Georgia, USA; n British Columbia Centre for Excellence in HIV/AIDS, Vancouver, British Columbia, Canada; o Division of AIDS, Faculty of Medicine, University of British Columbia, Vancouver, British Columbia, Canada; p Clinic of Infectious Diseases and Tropical Medicine, San Paolo Hospital, University of Milan, Milan, Italy; q Institute for Global Health, UCL, London, UK; r Service of Infectious Diseases, Lausanne University Hospital; s University of Lausanne, Lausanne, Switzerland; t Division of Infectious Disease, Department of Medicine, University of Alabama, Birmingham, Alabama; u Division of Infectious Diseases, Vanderbilt University Medical Center, Nashville, Tennessee, USA.

**Keywords:** CD4^+^ cell count, CD4^+^ : CD8^+^ ratio, CD8^+^ cell count, combination antiretroviral therapy, HIV

## Abstract

**Objective:**

Model trajectories of CD4^+^ and CD8^+^ cell counts after starting combination antiretroviral therapy (ART) and use the model to predict trends in these counts and the CD4^+^ : CD8^+^ ratio.

**Design:**

Cohort study of antiretroviral-naïve HIV-positive adults who started ART after 1997 (ART Cohort Collaboration) with more than 6 months of follow-up data.

**Methods:**

We jointly estimated CD4^+^ and CD8^+^ cell count trends and their correlation using a bivariate random effects model, with linear splines describing their population trends, and predicted the CD4^+^ : CD8^+^ ratio trend from this model. We assessed whether CD4^+^ and CD8^+^ cell count trends and the CD4^+^ : CD8^+^ ratio trend varied according to CD4^+^ cell count at start of ART (baseline), and, whether these trends differed in patients with and without virological failure more than 6 months after starting ART.

**Results:**

A total of 39 979 patients were included (median follow-up was 53 months). Among patients with baseline CD4^+^ cell count at least 50 cells/μl, predicted mean CD8^+^ cell counts continued to decrease between 3 and 15 years post-ART, partly driving increases in the predicted mean CD4^+^ : CD8^+^ ratio. During 15 years of follow-up, normalization of the predicted mean CD4^+^ : CD8^+^ ratio (to >1) was only observed among patients with baseline CD4^+^ cell count at least 200 cells/μl. A higher baseline CD4^+^ cell count predicted a shorter time to normalization.

**Conclusion:**

Declines in CD8^+^ cell count and increases in CD4^+^ : CD8^+^ ratio occurred up to 15 years after starting ART. The likelihood of normalization of the CD4^+^ : CD8^+^ ratio is strongly related to baseline CD4^+^ cell count.

## Abstract 2

Supplemental Digital Content is available in the text

## Introduction

Combination antiretroviral therapy (ART) has led to substantial increases in the life-expectancy of HIV-positive individuals [1]. Substantial declines in rates of AIDS have led to increased interest in non-AIDS, age-related diseases such as cardiovascular diseases, non-AIDS cancers, kidney disease and neurocognitive decline [2–7], rates of which are higher than in the general population [8]. HIV infection leads to persistent immune activation and inflammation, which may accelerate immunosenescence (deterioration of the immune system due to ageing) [9]. In the general population, a low CD4^+^ : CD8^+^ ratio is a surrogate marker for immunosenescence and an independent predictor of all-cause mortality [10,11]. Among HIV-positive individuals, low CD4^+^ : CD8^+^ ratio has been associated with higher levels of immunosenescence and inflammation, although the results regarding whether a low or inverted CD4^+^ : CD8^+^ ratio predicts non-AIDS-related morbidity and mortality have been conflicting [12,13].

The benefits of ART for recovery of CD4^+^ cell counts are well documented [14–16]. However, few studies have investigated long-term trends in CD8^+^ cell counts [17–21] and in CD4^+^ : CD8^+^ ratio [17–24] after starting ART, with almost all based on small (<150) or moderate (<2000) sample sizes. Given the potential health implications of elevated CD8^+^ cell counts and CD4^+^ : CD8^+^ ratios below 1 [19,20,25], more information is needed about their long-term trends in treated patients.

Our aims were to quantify long-term trends in CD8^+^ cell counts and CD4^+^ : CD8^+^ ratios, up to 15 years after starting ART, in a large cohort of antiretroviral-naïve individuals starting ART, and assess the impact of baseline CD4^+^ cell count on these trends.

## Methods

### Study patients

The ART Cohort Collaboration (ART-CC) is an international collaboration between prospective cohort studies of HIV-positive individuals residing in Europe and North America, described elsewhere [26] and at www.art-cohort-collaboration.org. Cohorts enrolled HIV-positive ART-naïve individuals aged at least 16 years starting treatment with a combination of at least three antiretroviral drugs. All cohorts provided anonymized data on a predefined set of demographic, laboratory and clinical variables, which were then pooled and analysed centrally. The NHS Health Research Authority South West, Cornwall and Plymouth Research Ethics Committee, UK, approved the study (REC reference 12/SW/0253).

Eligible patients were antiretroviral-naïve, started ART after 1997, had at least one CD4^+^ and CD8^+^ measurement within the baseline period (defined as 90 days before to 6 days after starting ART) and one or more CD4^+^ and CD8^+^ measurements 6 months after starting ART.

### Statistical analyses

CD4^+^ and CD8^+^ cell counts were natural-log transformed (zero counts were set to 1), to stabilize the variance and meet normality assumptions. When modelling the relationships of log-CD4^+^ and log-CD8^+^ with time, we considered fractional polynomials of one to four degrees with powers −2, −1, −0.5, 0, 0.5, 1, 2, 3 (power zero is interpreted as natural-log transformation) and linear spline models with two to five knots. We compared model fit using the Bayesian information criterion and the percentage of fitted values within 5% of observed values. We jointly modelled log-CD4^+^ and log-CD8^+^ using a bivariate random effects model. We included patient-level random effects for the intercept and trajectory terms, thus allowing trajectories to vary between patients. We allowed patient-level and measurement-level residuals to be correlated, thus allowing associations between CD4^+^ and CD8^+^ trajectories at both patient and measurement levels. Using the best fitting model, we estimated predicted means of log-CD4^+^ and log-CD8^+^, which when exponentiated became geometric means of CD4^+^ and CD8^+^ cell counts, respectively. We calculated the difference between predicted means of log-CD4^+^ and log-CD8^+^, which were exponentiated to derive predicted geometric mean CD4^+^ : CD8^+^ ratio. CD4^+^ : CD8^+^ ratios greater than 1 were defined as normalized.

Patients were classified by their baseline CD4^+^ (<25, 25–49, 50–99, 100–199, 200–349, 350–499, ≥500 cells/μl) and CD8^+^ (<500, 500–749, 750–999, ≥1000 cells/μl) cell counts. Patients with multiple measurements within the baseline period were classified using the measurement closest to their ART start date.

We included covariates sex, age at start of ART, risk transmission group, ethnicity and baseline CD4^+^ and CD8^+^ groups. To allow CD4^+^ and CD8^+^ trajectories to vary between baseline CD4^+^ groups, we included interactions between baseline CD4^+^ group and the intercept and trajectory terms.

Virological failure was defined as a HIV RNA measurement exceeding 1000 copies/ml, regardless of whether a patient had interrupted treatment. We generated a binary, time-independent variable denoting whether, from 6 months after starting ART, patients experienced at least one virological failure (virologically unsuppressed) or all viral load measurements were 1000 copies/ml or less (virologically suppressed). We predicted geometric means of CD4^+^ and CD8^+^ cell counts separately among patients classified as virologically suppressed and unsuppressed by including three-way interactions between the intercept and trajectory terms, baseline CD4^+^ group and viral suppression indicator.

In sensitivity analyses, we predicted geometric mean CD4^+^ and CD8^+^ cell counts separately among patients who started treatment before 2004 and from 2004 onwards, and included the 6% of patients who had no CD4^+^ or CD8^+^ measurements 6 months post-ART.

Analyses were conducted using STATA/MP, version 14 (StataCorp LLC, College Station, Texas, USA) [27], with the runmlwin command [28] to run software MLwiN, version 3.01 (Centre for Multilevel Modelling, University of Bristol, Bristol, UK) [29] from within STATA.

## Results

Of 110 098 patients included in ART-CC up to 31 December 2013, 15% were excluded as they started ART before 1998 or before entering the study, 43% without CD4^+^ or CD8^+^ measurements within the specified baseline period, and 6% as they had no CD4^+^ or CD8^+^ measurement after 6 months since starting ART. Table 1 presents characteristics of the remaining 39 979 eligible patients according to baseline CD4^+^ cell count. Most were men, approximately 40% were heterosexual and the median age at start of ART was about 40 years. Higher baseline CD4^+^ cell count predicted higher baseline CD8^+^ cell count, with substantial reductions in median CD8^+^ cell count as baseline CD4^+^ cell count decreased from 100–199 to less than 25 cells/μl.

**Table 1:** Characteristics of the 39 979 patients eligible for analysis.

	Baseline CD4^+^ cell count (cells/μl)						
	<25	25–49	50–99	100–199	200–349	350–499	≥500
Number of patients	2510	1779	3018	7455	14 141	6593	4483
Median (IQR) age (years)	38 (12)	40 (13)	39 (14)	39 (14)	38 (14)	38 (14)	37 (15)
Male (%)	76	75	73	70	72	73	70
Route of HIV infection (%)							
Homo/bisexual	27	31	33	34	41	45	43
IDU	11	11	11	11	9	9	9
Heterosexual	51	46	45	44	41	37	37
Other/not known	11	12	11	10	9	10	11
Median (IQR) baseline viral load (log_10_ copies/ml)	5.30 (0.79)	5.31 (0.81)	5.14 (0.85)	4.91 (0.94)	4.65 (1.00)	4.49 (1.35)	4.01 (2.57)
Median (IQR) baseline CD8^+^ (cells/μl)	340 (320)	480 (421)	610 (501)	749 (540)	900 (594)	981 (674)	1062 (715)
Median (IQR) follow-up (months)	64 (78)	64 (78)	61 (74)	62 (66)	50 (59)	41 (69)	47 (81)

IQR, interquartile range.

**Fig. 1:**
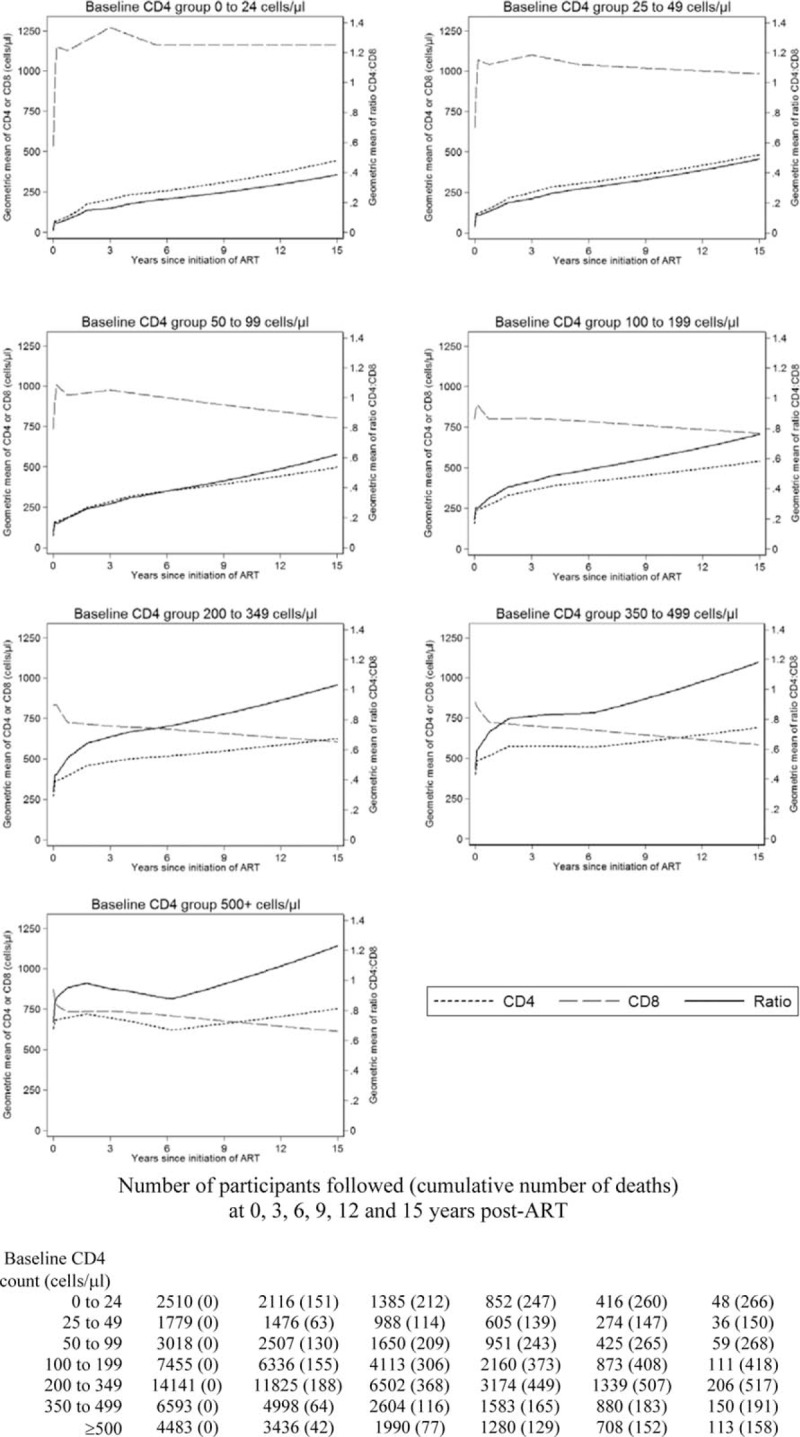
Predicted geometric mean CD4^+^ cell count trajectories, predicted geometric mean CD8^+^ cell count trajectories and derived predicted geometric mean CD4^+^ : CD8^+^ ratio trajectories, according to baseline CD4^+^ group.

Figure 1 shows trajectories of geometric mean CD4^+^ cell count, CD8^+^ cell count and CD4^+^ : CD8^+^ ratio according to baseline CD4^+^ group, predicted using the best fitting models (linear splines, with knots at 1, 21, 48 and 75 months for CD4^+^ cell count, and at 6 weeks, 9, 36 and 66 months for CD8^+^ cell count). Among patients with baseline CD4^+^ cell count less than 200 cells/μl, mean CD8^+^ cell counts increased steeply during the first 6 weeks of treatment, slowly decreased between 6 weeks and 9 months post-ART, slowly increased between 9 months and 3 years, and slowly decreased between 3 and 6 years. For example, among patients with baseline CD4^+^ cell count less than 25 cells/μl, the estimated ratios of geometric mean CD8^+^ cell count per month were 1.6724 (95% confidence interval 1.6509, 1.6938) during the first 6 weeks of treatment, 0.9971 (0.9948, 0.9994) between 6 weeks and 9 months post-ART, 1.0045 (1.0038, 1.0051) between 9 months and 3 years and 0.9969 (0.9963, 0.9975) between 3 and 6 years. Lower baseline CD4^+^ cell count predicted larger declines in CD8^+^ cell count between 3 and 6 years post-ART (refer to Appendix Table 1). Among patients with baseline CD4^+^ cell count 200–499 cells/μl, mean CD8^+^ cell counts slowly increased during the first 6 weeks, steeply decreased from 6 weeks to 9 months post-ART and then slowly decreased from 9 months post-ART. For example, among patients with baseline CD4^+^ cell count 200–349 cells/μl, the estimated ratios of geometric mean CD8^+^ cell count per month were 1.0076 (1.0022, 1.0130) during the first 6 weeks, 0.9805 (0.9795, 0.9814) between 6 weeks and 9 months, 0.9988 (0.9985, 0.9991) between 9 months and 3 years, 0.9993 (0.9991, 0.9996) between 3 and 6 years and 0.9989 (0.9986, 0.9991) from 6 years to end of follow-up. Among patients with baseline CD4^+^ cell count at least 500 cells/μl, mean CD8^+^ cell counts steeply decreased during the first 9 months post-ART, levelled-off between 9 months and 3 years, and then slowly decreased thereafter. From 6 years post-ART, mean CD8^+^ cell count plateaued among patients with baseline CD4^+^ cell count less than 50 cells/μl, but continued decreasing among patients with baseline CD4^+^ cell count at least 50 cells/μl.

Mean CD4^+^ cell counts increased throughout follow-up, except for patients with baseline CD4^+^ cell count at least 350 cells/μl in whom mean CD4^+^ cell counts plateaued or slowly decreased between 21 months and 6 years post-ART. Among all patients, the mean CD4^+^ : CD8^+^ ratio trajectory followed the same pattern as the mean CD4^+^ cell count, indicating that changes in CD4^+^ : CD8^+^ ratio were mainly driven by changes in CD4^+^ cell count. However, during periods of decreasing CD8^+^ cell count, relative increases in the mean CD4^+^ : CD8^+^ ratio were higher than those of mean CD4^+^ cell count. Among patients with baseline CD4^+^ cell count less than 50 cells/μl, increases in mean CD4^+^ : CD8^+^ ratio 6 years post-ART were only driven by CD4^+^ cell count increases, whereas among the remaining patients, increases in the mean CD4^+^ : CD8^+^ ratio were driven by both increasing CD4^+^ cell counts and decreasing CD8^+^ cell counts. During the 15-year follow-up period, the mean CD4^+^ : CD8^+^ ratio only exceeded 1 among patients with baseline CD4^+^ cell count at least 200 cells/μl: higher baseline CD4^+^ cell count predicted a shorter time to mean CD4^+^ : CD8^+^ ratio > 1.

Trends in mean CD8^+^ cell count and CD4^+^ : CD8^+^ ratio were similar between patients who were and were not virologically suppressed from 6 months post-ART (Appendix Tables 2 and 3). However, changes in mean CD8^+^ cell counts were more beneficial (smaller mean increases and larger mean decreases), and increases in the mean CD4^+^ : CD8^+^ ratio were larger, among virologically suppressed patients compared with those not suppressed.

Trends in mean CD4^+^ cell counts, CD8^+^ cell counts and CD4^+^ : CD8^+^ ratio were similar among patients who started treatment before 2004 and those who started 2004 onwards. However, patients starting treatment from 2004 onwards had higher mean CD4^+^ cell counts, lower mean CD8^+^ cell counts and higher CD4^+^ : CD8^+^ ratios throughout follow-up (results not shown). Including the 6% of patients who did not have any CD4^+^ or CD8^+^ measurements 6 months, post-ART had minimal effect on the results (results not shown).

## Discussion

Using data from a large collaborative study of HIV-infected individuals residing in Europe and North America, we found that, among patients with baseline CD4^+^ cell count at least 50 cells/μl, predicted mean CD8^+^ cell counts continued to decrease between 3 and 15 years post-ART, partly driving increases in the predicted mean CD4^+^ : CD8^+^ ratio. Lower baseline CD4^+^ cell count predicted higher increases in the mean CD4^+^ : CD8^+^ ratio during the first 6 years since start of ART. Nonetheless, even 15 years since start of ART, the mean CD4^+^ : CD8^+^ ratio did not normalize among patients with baseline CD4^+^ cell count less than 200 cells/μl.

Few studies have reported long-term trends in post-ART CD8^+^ cell counts. Two years post-ART, two studies reported similar findings to ours, of continued declines in CD8^+^ cell counts [18,21], whereas three studies reported stabilized CD8^+^ cell counts at elevated levels (approximately 600–900 cells/μl) [12,17,19]. However, sample sizes in these studies were small compared with ours (≤1253), and the patient population was heterogenous, consisting of late presenters or a mixture of treatment naïve and treatment experienced patients.

Our findings regarding the CD4^+^ : CD8^+^ ratio were similar to published small or restricted studies [18–20,23,24,30,31]. Continued increases in the CD4^+^ : CD8^+^ ratio for 8–15 years since start of treatment have been reported among patients with sustained undetectable viral loads [18,20,23]. Among patients with baseline CD4^+^ cell counts greater than 350 cells/μl, increases in the CD4^+^ : CD8^+^ ratio were partly attributed to decreases in CD8^+^ cell counts [18–20]. In one study examining CD4^+^ : CD8^+^ ratio trends by baseline CD4^+^ cell count, lower baseline CD4^+^ cell count predicted higher increases during the first 5 years after start of ART [18]. Higher baseline CD4^+^ cell count predicted faster time to normalized CD4^+^ : CD8^+^ ratio [30] and a greater likelihood of attaining a normalized ratio [18,24,30,31].

In conclusion, there are long-term decreases in CD8^+^ cell counts and long-term increases in CD4^+^ : CD8^+^ ratios, among patients who start ART with CD4^+^ cell count as low as 50–199 cells/μl. However, starting ART at high CD4^+^ cell counts is paramount for attainment of a maximal CD4^+^ : CD8^+^ ratio.

## Acknowledgements

R.A.H. was supported by Medical Research Council grant (MR/J013773/1). J.A.C.S. was supported by grant number MR/J002380/1: this award was jointly funded by the UK Medical Research Council (MRC) and the UK Department for International Development (DFID) under the MRC/DFID Concordat agreement and is also part of the EDCTP2 programme supported by the European Union. He was also supported by National Institute for Health Research Senior Investigator award NF-SI-0611-10168. V.D.L. is funded by a grant from the Canadian Institutes of Health Research (PJT-148595), by a Scholar Award from the Michael Smith Foundation for Health Research and a New Investigator award from the Canadian Institutes of Health Research. J.L.C. was funded by National Institutes of Health grant (NIAID K23AI120875).

### Conflicts of interest

D.C. reports potential conflicts of interest that are outside the submitted work. Grants from Janssen-Cilag (2014), Merck-Sharp & Dohme-Chibret (2017), ViiV (2015), personal fees from Janssen-Cilag (2016), Merck-Sharp & Dohme-Chibret (2015) for lectures, personal fees from Gilead (2014), ViiV (2015), Janssen-Cilag (2014) for travel/accommodations/meeting expenses, personal fees from Gilead France from 2011 until December 2015 for French HIV board, personal fees from Innavirvax (2015 and 2016) and Merck Switzerland (2017) for consultancy. P.R. through his institution has received independent scientific grant support from Gilead Sciences, Janssen Pharmaceuticals Inc., Merck & Co, and ViiV Healthcare; he has served on scientific advisory boards for Gilead Sciences, ViiV Healthcare, Merck and TEVA pharmaceutical industries for which his institution has received remuneration; he has also served on a data safety monitoring committee for Janssen Pharmaceuticals for which his institution has received remuneration. Remaining authors report no potential conflicts.

## Supplementary Material

Supplemental Digital Content
