# Supplementary material for: Long terms trends in CD4+ cell counts, CD8+ cell counts, and the CD4+ : CD8+ ratio
Source: AIDS. 2018 Jun 1;32(10):1361–7. doi: 10.1097/QAD.0000000000001848 (PMC5991182; doi:10.1097/QAD.0000000000001848)
Supplement: Supplemental Digital Content [file aids-32-1361-s001.docx]

Appendix table 1: Estimated ratios of predicted geometric means of CD4 counts comparing year 3 post combination antiretroviral therapy (post-ART) with year 0, year 6 with year 3, and year 9 with year 6, according to baseline CD4 count. The same comparisons for estimated ratios of predicted geometric means of CD8 counts and the geometric CD4:CD8 ratio. 95% confidence intervals displayed within brackets.

| Baseline  CD4 count  cells/mm^3^ | 0 to 3 years post-ART | | |  | 3 to 6 years post-ART | | |  | 6 to 9 years post-ART | | |
| --- | --- | --- | --- | --- | --- | --- | --- | --- | --- | --- | --- |
|  | CD4 count | CD8 count | Ratio CD4:CD8 |  | CD4 count | CD8 count | Ratio CD4:CD8 |  | CD4 count | CD8 count | Ratio CD4:CD8 |
| 0 to 24 | 18.1*  [17.6, 18.5] | 2.39  [2.33, 2.44] | 7.57  [7.37, 7.78] |  | 1.26  [1.23, 1.29] | 0.91  [0.90, 0.93] | 1.38  [1.35, 1.41] |  | 1.20  [1.17, 1.23] | 1.00  [0.98, 1.02] | 1.20  [1.17, 1.22] |
| 25 to 49 | 6.55  [6.36, 6.74] | 1.69  [1.64, 1.73] | 3.88  [3.76, 4.01] |  | 1.24  [1.21, 1.28] | 0.94^$^  [0.92, 0.96] | 1.32  [1.28, 1.36] |  | 1.16  [1.13, 1.19] | 0.98  [0.96, 1.00] | 1.18  [1.15, 1.21] |
| 50 to 99 | 3.79  [3.70, 3.87] | 1.32  [1.30, 1.35] | 2.86  [2.79, 2.93] |  | 1.23  [1.20, 1.26] | 0.95  [0.94, 0.97] | 1.29  [1.26, 1.32] |  | 1.13  [1.10, 1.15] | 0.95  [0.94, 0.97] | 1.18^#^  [1.16, 1.21] |
| 100 to 199 | 2.29  [2.26, 2.33] | 1.00  [0.99, 1.01] | 2.29  [2.26, 2.33] |  | 1.15  [1.13, 1.17] | 0.98  [0.97, 0.99] | 1.18  [1.16, 1.19] |  | 1.09  [1.08, 1.11] | 0.97  [0.96, 0.98] | 1.13  [1.12, 1.15] |
| 200 to 349 | 1.78  [1.76, 1.80] | 0.84  [0.84, 0.85] | 2.11  [2.09, 2.13] |  | 1.07  [1.06, 1.08] | 0.97  [0.97, 0.98] | 1.10  [1.09, 1.11] |  | 1.06  [1.05, 1.08] | 0.96  [0.95, 0.97] | 1.11  [1.10, 1.12] |
| 350 to 499 | 1.44  [1.41, 1.46] | 0.82  [0.81, 0.84] | 1.74  [1.71, 1.77] |  | 0.99  [0.97, 1.01] | 0.97  [0.95, 0.98] | 1.02  [1.00, 1.04] |  | 1.06  [1.05, 1.08] | 0.95  [0.94, 0.96] | 1.12  [1.10, 1.13] |
| 500 | 1.11  [1.08, 1.13] | 0.84  [0.83, 0.86] | 1.31  [1.28, 1.34] |  | 0.90  [0.88, 0.92] | 0.96  [0.95, 0.98] | 0.93  [0.91, 0.95] |  | 1.05  [1.03, 1.07] | 0.95  [0.94, 0.96] | 1.11  [1.09, 1.13] |

* Interpretation of geometric ratio: Among patients with baseline CD4 count 0 to 24 cells/mm^3^, geometric mean CD4 count at 3 years was just over 18 times higher than the geometric mean CD4 count at start of ART.

$ Interpretation of geometric ratio: Among patients with baseline CD4 count 25 to 49 cells/mm^3^, geometric mean CD8 count at 6 years post-ART was 6% lower compared to the geometric mean CD8 count at 3 years post-ART.

# Interpretation of geometric ratio: Among patients with baseline CD4 count 50 to 99 cells/mm^3^, geometric CD4:CD8 ratio at 9 years post-ART was 18% higher than the geometric CD4:CD8 ratio at 6 years post-ART.

Appendix table 2: Among patients virologically suppressed (≤1000 copies/mL) from 6 months after starting combination antiretroviral therapy, estimated ratios of predicted geometric means of CD4 counts comparing year 3 post-ART with year 0, year 6 with year 3, and year 9 with year 6, according to baseline CD4 count. The same comparisons for estimated ratios of predicted geometric means of CD8 counts and the geometric CD4:CD8 ratio. 95% confidence intervals displayed within brackets.

| Baseline  CD4 count  cells/mm^3^ | 0 to 3 years post-ART | | |  | 3 to 6 years post-ART | | |  | 6 to 9 years post-ART | | |
| --- | --- | --- | --- | --- | --- | --- | --- | --- | --- | --- | --- |
|  | CD4 count | CD8 count | Ratio CD4:CD8 |  | CD4 count | CD8 count | Ratio CD4:CD8 |  | CD4 count | CD8 count | Ratio CD4:CD8 |
| 0 to 24 | 25.7*  [24.9, 26.5] | 2.47  [2.40, 2.55] | 10.4  [10.1, 10.7] |  | 1.30  [1.26, 1.35] | 0.87  [0.85, 0.90] | 1.49  [1.44, 1.54] |  | 1.15  [1.12, 1.19] | 0.99  [0.96, 1.01] | 1.17  [1.13, 1.21] |
| 25 to 49 | 8.49  [8.18, 8.79] | 1.71  [1.65, 1.77] | 4.96  [4.76, 5.15] |  | 1.27  [1.22, 1.32] | 0.91^$^  [0.88, 0.94] | 1.39  [1.34, 1.45] |  | 1.15  [1.11, 1.19] | 0.98  [0.95, 1.00] | 1.18  [1.14, 1.22] |
| 50 to 99 | 4.64  [4.52, 4.77] | 1.33  [1.29, 1.36] | 3.49  [3.39, 3.59] |  | 1.26  [1.22, 1.29] | 0.93  [0.91, 0.95] | 1.35  [1.31, 1.39] |  | 1.10  [1.07, 1.13] | 0.94  [0.92, 0.96] | 1.17^#^  [1.13, 1.20] |
| 100 to 199 | 2.64  [2.59, 2.68] | 0.97  [0.95, 0.98] | 2.72  [2.68, 2.77] |  | 1.20  [1.18, 1.23] | 0.97  [0.95, 0.98] | 1.24  [1.22, 1.27] |  | 1.08  [1.06, 1.10] | 0.96  [0.95, 0.98] | 1.12  [1.10, 1.15] |
| 200 to 349 | 1.98  [1.96, 2.00] | 0.80  [0.79, 0.81] | 2.48  [2.45, 2.52] |  | 1.13  [1.11, 1.14] | 0.96  [0.95, 0.97] | 1.17  [1.15, 1.19] |  | 1.05  [1.04, 1.07] | 0.96  [0.95, 0.97] | 1.10  [1.08, 1.12] |
| 350 to 499 | 1.63  [1.59, 1.66] | 0.76  [0.74, 0.77] | 2.14  [2.10, 2.19] |  | 1.08  [1.05, 1.11] | 0.95  [0.93, 0.97] | 1.14  [1.11, 1.17] |  | 1.03  [1.00, 1.05] | 0.94  [0.92, 0.96] | 1.09  [1.06, 1.12] |
| 500 | 1.27  [1.23, 1.30] | 0.77  [0.75, 0.79] | 1.65  [1.60, 1.69] |  | 1.04  [1.00, 1.07] | 0.95  [0.93, 0.98] | 1.09  [1.05, 1.13] |  | 1.02  [0.99, 1.06] | 0.95  [0.93, 0.97] | 1.08  [1.04, 1.11] |

* Interpretation of geometric ratio: Among virologically suppressed patients with baseline CD4 count 0 to 24 cells/mm^3^, geometric mean CD4 count at 3 years was close to 26 times higher than the geometric mean CD4 count at start of ART.

$ Interpretation of geometric ratio: Among virologically suppressed patients with baseline CD4 count 25 to 49 cells/mm^3^, geometric mean CD8 count at 6 years post-ART was 9% lower compared to the geometric mean CD8 count at 3 years post-ART.

# Interpretation of geometric ratio: Among virologically suppressed patients with baseline CD4 count 50 to 99 cells/mm^3^, geometric CD4:CD8 ratio at 9 years post-ART was 17% higher than the geometric CD4:CD8 ratio at 6 years post-ART.

Appendix table 3: Among patients who experienced a virological failure (>1000 copies/mL) from 6 months after starting combination antiretroviral therapy (ART), estimated ratios of predicted geometric means of CD4 counts comparing year 3 post-ART with year 0, year 6 with year 3, and year 9 with year 6, according to baseline CD4 count. The same comparisons for estimated ratios of predicted geometric means of CD8 counts and the geometric CD4:CD8 ratio. 95% confidence intervals displayed within brackets.

| Baseline  CD4 count  cells/mm^3^ | 0 to 3 years post-ART | | |  | 3 to 6 years post-ART | | |  | 6 to 9 years post-ART | | |
| --- | --- | --- | --- | --- | --- | --- | --- | --- | --- | --- | --- |
|  | CD4 count | CD8 count | Ratio CD4:CD8 |  | CD4 count | CD8 count | Ratio CD4:CD8 |  | CD4 count | CD8 count | Ratio CD4:CD8 |
| 0 to 24 | 11.5*  [11.1, 11.9] | 2.28  [2.20, 2.35] | 5.04  [4.85, 5.23] |  | 1.26  [1.21, 1.30] | 0.96  [0.93, 0.98] | 1.31  [1.27, 1.36] |  | 1.27  [1.23, 1.31] | 1.02  [1.00, 1.04] | 1.25  [1.21, 1.29] |
| 25 to 49 | 4.58  [4.39, 4.78] | 1.66  [1.59, 1.73] | 2.76  [2.64, 2.89] |  | 1.24  [1.19, 1.29] | 0.98^$^  [0.95, 1.01] | 1.27  [1.22, 1.32] |  | 1.19  [1.14, 1.23] | 0.99  [0.97, 1.02] | 1.20  [1.15, 1.24] |
| 50 to 99 | 2.75  [2.65, 2.84] | 1.31  [1.27, 1.35] | 2.10  [2.02, 2.18] |  | 1.22  [1.18, 1.26] | 0.98  [0.96, 1.01] | 1.24  [1.20, 1.28] |  | 1.18  [1.14, 1.21] | 0.97  [0.95, 0.99] | 1.22^#^  [1.18, 1.26] |
| 100 to 199 | 1.78  [1.74, 1.82] | 1.06  [1.04, 1.09] | 1.68  [1.64, 1.72] |  | 1.09  [1.07, 1.12] | 0.99  [0.97, 1.00] | 1.11  [1.08, 1.13] |  | 1.13  [1.11, 1.15] | 0.98  [0.96, 0.99] | 1.16  [1.14, 1.18] |
| 200 to 349 | 1.41  [1.38, 1.43] | 0.96  [0.94, 0.98] | 1.46  [1.44, 1.49] |  | 1.01  [0.99, 1.03] | 0.98  [0.97, 0.99] | 1.03  [1.01, 1.05] |  | 1.10  [1.09, 1.12] | 0.96  [0.95, 0.97] | 1.15  [1.13, 1.17] |
| 350 to 499 | 1.18  [1.15, 1.21] | 0.95  [0.93, 0.97] | 1.24  [1.21, 1.28] |  | 0.93  [0.91, 0.95] | 0.97  [0.95, 0.99] | 0.96  [0.94, 0.98] |  | 1.11  [1.09, 1.13] | 0.96  [0.95, 0.97] | 1.15  [1.13, 1.18] |
| 500 | 0.96  [0.94, 0.99] | 0.94  [0.91, 0.96] | 1.03  [1.00, 1.06] |  | 0.84  [0.82, 0.86] | 0.96  [0.94, 0.98] | 0.88  [0.86, 0.90] |  | 1.08  [1.06, 1.11] | 0.95  [0.94, 0.96] | 1.14  [1.12, 1.16] |

* Interpretation of geometric ratio: Among patients who experienced virological failure with baseline CD4 count 0 to 24 cells/mm^3^, geometric mean CD4 count at 3 years was close to 12 times higher than the geometric mean CD4 count at start of ART.

$ Interpretation of geometric ratio: Among patients who experienced virological failure with baseline CD4 count 25 to 49 cells/mm^3^, geometric mean CD8 count at 6 years post-ART was 2% lower compared to the geometric mean CD8 count at 3 years post-ART.

# Interpretation of geometric ratio: Among patients who experienced virological failure with baseline CD4 count 50 to 99 cells/mm^3^, geometric CD4:CD8 ratio at 9 years post-ART was 22% higher than the geometric CD4:CD8 ratio at 6 years post-ART.
